# Urinary Cadmium and Osteoporosis in U.S. Women ≥50 Years of Age: NHANES 1988–1994 and 1999–2004

**DOI:** 10.1289/ehp.11452

**Published:** 2008-06-13

**Authors:** Carolyn M. Gallagher, John S. Kovach, Jaymie R. Meliker

**Affiliations:** 1 Graduate Program in Public Health; 2 Department of Preventive Medicine, Stony Brook University Medical Center, Stony Brook, New York, USA

**Keywords:** bone mineral density, cadmium, femur, hip, osteoporosis, women

## Abstract

**Background:**

Urinary cadmium (U-Cd) has been associated with decreased peripheral bone mineral density (BMD) and osteoporosis. This association, however, has not been confirmed using femoral BMD, the international standard for diagnosing osteoporosis, at levels < 1.0 μg Cd/g creatinine.

**Objectives:**

Our goal was to investigate the statistical association between U-Cd, at levels ≤ 1 μg/g creatinine, and osteoporosis, as indicated by hip BMD and self-report in a population-based sample of U.S. women ≥ 50 years of age.

**Methods:**

We drew data from the National Health and Nutrition Examination Surveys for 1988–1994 (*n* = 3,207) and 1999–2004 (*n* = 1,051). Osteoporosis was indicated by hip BMD cutoffs based on the international standard and self-report of physician diagnosis. We analyzed U-Cd levels for association with osteoporosis using multiple logistic regression.

**Results:**

Women ≥ 50 years of age with U-Cd levels between 0.50 and 1.00 μg/g creatinine were at 43% greater risk for hip-BMD–defined osteoporosis, relative to those with levels ≤ 0.50 μg/g (odds ratio = 1.43; 95% confidence interval, 1.02–2.00; *p* = 0.04). We observed similar effect estimates using self-report of physician-diagnosed osteoporosis. Smokers did not show a statistically increased risk.

**Conclusions:**

Results suggest that U.S. women are at risk for osteoporosis at U-Cd levels below the U.S. Occupational Safety and Health Administration’s 3-μg/g safety standard. Given null findings among smokers, dietary Cd, rather than tobacco, is the likely source of Cd-related osteoporosis risk for the U.S. female population ≥ 50 years of age.

Osteoporosis, a deterioration of bone tissue that results in low bone mineral density (BMD) and risk of fracture [World Health Organization (WHO) 2003], poses a disease burden that exceeds that of hypertension and breast cancer (WHO 2004). The Joint Food and Agricultural Organization of the United Nations/WHO Expert Committee on Food Additives (JECFA) recently concluded that substantial uncertainty remains regarding the long-term significance of the effects of cadmium on bone (JECFA 2005), citing mixed results from epidemiologic studies that investigated the association between low-level environmental exposure to Cd and its direct effects on bone.

Cadmium is a toxic metal that is released into the environment from industrial activity, including mining and smelting, fuel combustion (e.g., coal-fired power plants), disposal of metal-containing products, and application of municipal sludge or phosphate fertilizer [Agency for Toxic Substances and Disease Registry (ATSDR) 1999]. Human exposure to Cd is primarily through food [International Programme on Chemical Safety (IPCS) 1992], with low levels of Cd found in all foods (ATSDR 1999). Smokers may have up to twice the Cd intake compared with non-smokers because cigarette smoke contains Cd taken up by the tobacco plant (ATSDR 1999). The U.S. Food and Drug Administration’s Total Diet Study update reported a 26% increase in dietary Cd exposure from 1990 through 2003, from 8.81 to 11.06 μg/person/day; the latter exposure constitutes 21% of the provisional tolerable weekly intake (PTWI) (Egan et al. 2007).

Cadmium accumulates in the human body, particularly the kidney, where it can remain for many years. A small portion of Cd is slowly excreted in urine (ATSDR 1999). Urinary Cd (U-Cd) is a biomarker for lifetime Cd body burden in people with lower exposures because, in the absence of episodes of high-level exposure, Cd-binding sites are not saturated, and the urine Cd level increases in proportion to the amount of Cd stored in the body [Centers for Disease Control and Prevention (CDC) 2005; IPCS 1992].

U-Cd has been inversely associated with forearm BMD in studies from Sweden and Belgium (Akesson et al. 2006; Alfven et al. 2000; Staessen et al. 1999); however, mixed results were reported in Japan. Honda et al. (2003) found that ultrasound-measured stiffness index, an index of calcaneal bone mass, was significantly inversely correlated with U-Cd, in the absence of kidney damage, whereas Horiguchi et al. (2005) reported no association between Cd and bone effects after adjustment for renal effects. Tubular renal dysfunction has been found at U-Cd levels as low as 1 μg/g creatinine (Jarup et al. 2000). Chen et al. (2006) investigated the relationship between U-Cd and β_2_-microglobulinuria, a biomarker of Cd-induced renal tubular damage, in a type 2 diabetic population and showed statistically significant increased odds of tubular renal dysfunction at U-Cd levels ≥1 μg/g creatinine relative to U-Cd levels <1.0 μg/g creatinine. Akesson et al. (2006) reported associations between increasing overall Cd levels and decreasing forearm BMD and a direct effect on bone, that is, increasing bone resorption. They did not, however, compare risks for osteoporosis associated with specific Cd levels. The Osteoporosis-Cadmium as a Risk Factor (OSCAR) study (Jarup and Alfven 2004) identified a dose–response relationship between U-Cd 3 μg/g creatinine and higher and low BMD, but additional studies are needed to clarify the risk of osteoporosis associated with widespread exposure and U-Cd at levels < 1.0 g/g creatinine associated with renal tubular dysfunction. Additionally, these studies used peripheral bone density measures, whereas the international reference standard is based on a femoral neck BMD measure (WHO 2004). Moreover, fracture rates vary substantially worldwide (WHO 2004), and there is a dearth of evidence concerning the association between Cd and osteoporosis in North American populations.

Our primary objective was to investigate the association between U-Cd, particularly at levels ≤ 1.0 μg/g creatinine, and hip BMD at levels indicative of osteoporosis according to WHO criterion in a population-based sample of U.S. women ≥ 50 years of age. Our secondary objective was to investigate the associations between U-Cd and survey-respondent–reported physician diagnosis of osteoporosis, and to compare the results of the two outcome measures. We hypothesized that higher levels of U-Cd might be associated with greater odds for osteoporosis, and that Cd levels between 0.50 and 1.0 μg/g creatinine might suggest a significant exposure–effect relationship relative to the reference group with the lowest Cd levels.

## Materials and Methods

We obtained the study sample data sets from the Third U.S. National Health and Nutrition Examination Survey (NHANES III) 1988–1994 survey data (CDC 2008) for the primary analysis of hip BMD, and NHANES 1999–2004 data (CDC 2007a, 2007b, 2007c) for the secondary analysis of survey-respondent–reported physician diagnosis of osteoporosis. NHANES is a cross-sectional, random household survey of the civilian population based on a complex probability sampling design (CDC 2006). NHANES 1988–1994 data sets included two phases of data each obtained from interviews of 33,994 participants and examinations of 30,818 participants (CDC 1996a). BMD measurements were obtained by bone densitometry, conducted at the Medical Examination Center and performed on 14,646 men and women ≥ 20 years of age. We limited our analysis to women ≥ 50 years of age because female sex and age of ≥ 50 years are recognized risk factors for osteoporosis (Dontas and Yiannakpoulos 2007). BMD measurements were performed on 3,379 women ≥ 50 years of age, with 2% rejected and 3,311 with acceptable BMD data (Looker et al. 1997). The female response rate for BMD examination was 82.43% (Mohadjer et al. 1994). Overall, the ranges of interviewed and examined sample response rates for women ≥ 50 years of age were 78–81% and from 51–73%, respectively, for NHANES III [National Center for Health Statistics (NCHS) 2008]. NHANES 1999–2000, 2001–2002, and 2003–2004 data sets included a total of 31,126 participants (CDC 2007a, 2007b, 2007c). The ranges of female interviewed and examined response rates for women ≥ 50 years of age were 71–75% and 52–71%, respectively, for NHANES 1999–2000; 72–77% and 53–74%, respectively, for NHANES 2001–2002; and 67–77% and 57–72%, respectively, for NHANES 2003–2004 (NCHS 2008). The lowest response rates, that is, 51–57%, were for examined women ≥ 80 years of age.

For the primary analysis, osteoporosis status was indicated by either femur neck BMD < 0.56 g/cm^2^ or total hip BMD < 0.64 g/cm^2^, per the osteoporosis cutoffs reported in the NHANES III femoral bone density study conducted by Looker et al. (1997), and consistent with the international reference standard (WHO 2004). The international reference standard is used worldwide to diagnose osteoporosis at the hip and is based on a femoral neck BMD of ≥ 2.5 SDs below the young female adult mean (CDC 2007d). For the secondary analysis, osteoporosis status was indicated by survey participant reporting during the NHANES interview. The interview question read, “Has a doctor ever told you that you had osteoporosis, sometimes called thin or brittle bones?”

For both primary and secondary analyses, urinary Cd was obtained from a single urine specimen collected and measured by the CDC laboratory using atomic absorption spectrometry and reported in nanograms per milliliter. Urinary creatinine was obtained from the same urine specimen and measured in milligrams per deciliter. Quality control procedures call for urine specimens to be processed, stored, and shipped to the CDC laboratory for analysis per NCHS quality control protocol (CDC 1996b, 2007e). A creatinine-adjusted U-Cd measure (micrograms per gram) was generated by dividing U-Cd (nanograms per milliliter) by urinary creatinine (milligrams per deciliter). Analyses were limited to women with CDC laboratory-measured Cd and creatinine, with U-Cd levels ≤ 20 ng/mL. In an NHANES II study, Whittemore et al. (1991) used this same 20-ng/mL exclusion, citing the determination by Friberg et al. (1985) that 20 μg/L (ng/mL) is considered an upper bound for plausible values in environmental exposures. As a result, no observations were excluded in our primary analysis, and only one observation, with an extreme creatinine-adjusted Cd level of 282.90 μg/g, was excluded in the secondary analysis. To facilitate comparisons with the OSCAR study, which used U-Cd levels of < 0.50, 0.50 to < 3.00, and ≥3.00 nmol/mmol creatinine (Alfven et al. 2000), with 1 nmol/mmol creatinine = 1 μg/g creatinine (Jarup et al. 2000), we used a reference U-Cd level of ≤ 0.50 μg/g creatinine. However, to investigate relative effects at lower levels of Cd (i.e., < 1.0 μg/g creatinine), we used the following U-Cd levels: > 0.50–1.00 μg/g creatinine and > 1.00 μg/g creatinine.

In our primary analysis, 664 women > 50 years of age had osteoporosis and 2,543 did not; in our secondary analysis, 200 women in this age group had osteoporosis and 851 did not. We conducted logistic regression to calculate the urinary creatinine-adjusted Cd odds ratio (OR) for osteoporosis, both unadjusted and adjusted for age, race (white compared with nonwhite), family income category (lowest to highest), underweight (< 127 lb or 57.61 kg), and smoking status. According to the National Osteoporosis Foundation’s *Physician’s Guide to Prevention and Treatment of Osteoporosis* (National Osteoporosis Foundation 2003), low body weight (e.g., ~ 127 lb) is a major risk factor for osteoporosis and related fracture in Caucasian post-menopausal women. We delineated smoking status into ever-smokers, who reported having smoked at least 100 cigarettes and other tobacco in their lifetime and/or current smoking every day or some days, versus never-smokers, who responded negatively to both former and current smoking.

We also adjusted for reported renal impairment in the secondary analysis in an effort to proxy Cd-induced renal tubular damage that may indirectly result in low BMD and osteoporosis through secondary effects on bone (Chen et al. 2006; Jarup et al. 2000). A surrogate measure was available in the NHANES 1999–2004 survey data sets: We defined impaired renal function as a “yes” response to the NHANES survey question, “Have you ever been told by a doctor or other health professional that you had weak or failing kidneys? Do not include kidney stones, bladder infections, or incontinence.” The same question was not asked in the NHANES III study, nor were biomarkers of Cd-induced renal tubular damage available, for example, urinary retinol-binding protein (Jarup et al. 2000; Schutte et al. 2008) or β_2_-microglobulin (Chen et al. 2006). Consequently, the primary analysis did not adjust for renal impairment.

We also investigated the relationship between U-Cd and BMD from NHANES III using multiple linear regression. We conducted this analysis to compare results using continuous BMD measures with those relying on BMD cutoffs of osteoporosis (Looker et al. 1997), as are used in multiple logistic regression analyses.

We conducted statistical analysis for calculation of ORs using SAS, version 9.1 (SAS Institute Inc., Cary, NC). We used SAS-callable Sudaan to calculate 95% confidence intervals (95% CIs) and *p*-values (of Satterwaithe adjusted *F*-test statistics) for all statistical analyses, as well as β-coefficients and multiple *R*^2^ values for linear regression, per NHANES analytic guidelines (CDC 1996b, 2006). Also in accordance with NHANES analytic guidelines for complex survey analysis of one or more variables from the medical examination component (MEC) (i.e., U-Cd and creatinine), we used NHANES III MEC weights (Looker et al. 1997) and NHANES 6-year MEC weights (CDC 2006). We calculated 6-year MEC weights for the appropriate data release years using 4-year MEC weights and 2-year MEC weights. The convergence criterion was satisfied for logistic regression analyses, and global chi-square statistics were significant, thus providing no indication to question the validity of model fit. In the linear regression analyses, we analyzed two dependent variables in separate regression equations: femur BMD and total hip BMD. Plots of both dependent variables approximated normal distributions; however, plots of creatinine-adjusted U-Cd did not, so we standardized creatinine-adjusted U-Cd by natural log transformation of its value + 1.

## Results

### Unadjusted findings

The arithmetic mean U-Cd for U.S. women 50–90 years of age in the NHANES 1988–1994 sample (with hip BMD measured) was 0.96, with a maximum of 16.17 μg/g creatinine. Mean U-Cd was higher for women with osteoporosis (1.12 μg/g) than for those without osteoporosis (0.92 μg/g). For a 1-μg/g increase in U-Cd, a woman had 38% greater odds for osteoporosis (OR = 1.38; *p*-value < 0.01), unadjusted for demographic and health characteristics. Women with Cd levels between 0.50 and 1.0 μg/g creatinine had 55% greater odds for osteoporosis compared with women with levels ≤ 0.50 μg/g (OR = 1.55; *p* < 0.01; Table 1).

In the NHANES 1999–2004 sample (with respondent-reported physician diagnosis of osteoporosis), mean U-Cd for women 50–85 years of age was 0.63 μg/g, with a maximum of 4.20 μg/g. Mean U-Cd was higher for women with osteoporosis (0.69 μg/g) compared with women without osteoporosis (0.62 μg/g). For a 1-μg/g increase in U-Cd, a woman had 59% greater odds for osteoporosis, unadjusted for demographic and health characteristics (OR = 1.59; *p* = 0.02). Effects at Cd levels between 0.50 and 1.0 μg/g creatinine were similar to those in the BMD analysis (OR = 1.60; *p* = 0.04; Table 2).

### Adjusted findings, multiple logistic regression

In this statistical analysis, osteoporosis was the dependent variable and creatinine-adjusted U-Cd was the primary predictor. For a 1-μg/g creatinine increase in U-Cd, women ≥ 50 years of age had 15% greater odds for osteoporosis (OR = 1.15; 95% CI, 1.00–1.33; *p* = 0.05), as defined by hip BMD, adjusted for age (OR = 1.09; 95% CI, 1.07–1.11; *p* < 0.01), white race (OR = 1.35; 95% CI, 0.95–1.92; *p* = 0.09), income (OR = 1.00; 95% CI, 0.98–1.02; *p* = 0.80), ever-smoker (OR = 1.17; 95% CI, 0.85–1.61; *p* = 0.33), and underweight (OR = 6.70; 95% CI, 4.48–10.02; *p* < 0.01). Compared with the reference group (≤ 0.50 μg Cd/g creatinine), women ≥ 50 years of age with U-Cd levels between 0.50 and 1.0 μg/g had 43% greater odds for osteoporosis (OR = 1.43; 95% CI, 1.02–2.00; *p* = 0.04), as measured by hip BMD, adjusted for age (OR = 1.09; 95% CI, 1.07–1.11; *p* < 0.01), white race (OR = 1.32; 95% CI, 0.92–1.88; *p* = 0.13), income (OR = 1.00; 95% CI, 0.98–1.02; *p* = 0.69), ever smoker (OR = 1.15; 95% CI, 0.83–1.58; *p* = 0.40), and underweight (OR = 6.85; 95% CI, 4.52–10.40; *p* < 0.01; Table 3).

The corresponding OR was similar for women who reported a diagnosis of osteoporosis (OR = 1.46), and although statistical significance was not evident at Cd levels between 0.50 and 1.00 μg/g for this outcome measure, overall incremental Cd was significantly associated with osteoporosis (OR = 1.65; 95% CI, 1.07–2.54; *p* = 0.02), adjusted for age (OR = 1.05; 95% CI, 1.03–1.07; *p* < 0.01), white race (OR = 2.58, 95% CI, 1.44–4.62; *p* < 0.01), income (OR = 0.98, 95% CI, 0.90–1.07; *p* = 0.69), ever-smoker (OR = 1.16; 95% CI, 0.72–1.86; *p* = 0.54), underweight (OR = 1.67; 95% CI, 1.08–2.57; *p* = 0.02), and renal disease (OR = 2.89; 95% CI, 1.21–6.87; *p* = 0.02; Table 3). Adjusted findings for ever-smokers did not indicate a statistically significant risk per 1-μg/g creatinine increment in U-Cd, although a marginally significant risk was evident for never-smokers in the NHANES 1999–2004 analysis (OR = 2.05; 95% CI, 0.99–4.23; *p* = 0.05; Table 4).

### Adjusted findings, multiple linear regression

In these statistical analyses, BMD (separately for femur and total hip) was the dependent variable and the natural log of creatinine-adjusted U-Cd was the primary predictor. The natural log of creatinine-adjusted U-Cd showed statistically significant inverse associations with femur BMD (β = −0.04; 95% CI, −0.06 to −0.02; *p* < 0.01) and with total hip BMD (β = −0.05; 95% CI, −0.07 to −0.03; *p* < 0.01). Age, white race, and underweight were statistically significant covariates for both outcomes, whereas smoking and income were not statistically significant (Table 5).

## Discussion

In the present study, our findings of statistically significant associations between Cd exposure and osteoporosis, and between Cd and BMD, in the U.S. population are consistent with previously reported studies of populations in Sweden (Akesson et al. 2006; Alfven et al. 2000) Belgium (Staessen et al. 1999), and Japan (Honda et al. 2003). However, an important distinction is that, in this study of the U.S. female population ≥ 50 years of age, U-Cd was significantly associated with greater odds for hip-BMD–defined osteoporosis at levels ≤ 1.0 μg/g creatinine. Adjusted Cd levels < 1.00 μg/g were not significantly associated with respondent-reported physician-diagnosed osteoporosis in the NHANES 1999–2004 data set, although the effect estimates were highly similar (OR ≈ 1.40) across both NHANES data sets. Osteoporosis prevalence was consistent across both NHANES data sets at 20%. The smaller sample size of the NHANES 1999–2004 data set may have limited our ability to detect statistical significance of adjusted Cd levels < 1.00 μg/g for reported osteoporosis.

Findings were consistent with our primary hypothesis that Cd levels between 0.50 and 1.0 μg/g creatinine are associated with osteoporosis, relative to reference group levels of ≤ 0.50 μg/g creatinine. Additionally, osteoporosis and BMD were statistically associated with age and underweight, but not with smoking status or income. Self-reported renal impairment was statistically associated with self-reported osteoporosis, as well. We found evidence of risk of Cd-related osteoporosis at levels less than half that of the OSCAR study (Jarup and Alfven 2004). Our ability to detect risks from lower level exposures reflects that our population resided in areas of no known Cd contamination, in contrast to the OSCAR study population (Jarup and Alfven 2004). In opposition to our findings, Horiguchi et al. (2005) reported no association between U-Cd and BMD in female Japanese farmers. One possible explanation is selection bias: those women able to continue to work as farmers and thus be eligible for Horiguchi et al.’s study were likely not to have the debilitating effects of osteoporosis. Another design flaw is the lack of transparent and objective selection criteria for designated contaminated and uncontaminated populations (Horiguchi et al. 2005).

Our study has several advantages over previous studies. First, we used decreased hip BMD measurements based on the WHO international standard (Looker et al. 1997), rather than on peripheral measurements. Second, we found statistically increased osteoporosis risk at Cd levels < 1.00 μg Cd/g creatinine in a large, varied sample representative of a population with widespread exposure. Finally, we showed similar effects for a subsequent population-representative sample reporting physician-diagnosed osteoporosis in the NHANES 1999–2004 data set.

The primary and secondary analyses have complementary strengths and weaknesses. Lack of confirmation of respondent-reported diagnostic findings can be interpreted as a limitation to our analysis of NHANES 1999–2004 data. However, our analysis of NHANES III BMD-based data may be limited by the lack of expert consensus on the use of BMD as a diagnostic criterion for osteoporosis (CDC 2007d; National Institutes of Health 2000). Specificity is limited; that is, a physician cannot definitively rule out osteoporosis at other sites (Nelson et al. 2002). Moreover, Cummings et al. (2002) asserted that the relationship between decreased BMD and increased risk of fracture is continuous so that a cutoff value to differentiate high-risk from low-risk individuals is problematic. Results of multiple linear regression analyses that took advantage of continuous BMD, however, also revealed an inverse association between BMD and U-Cd, lending support to the logistic regression analyses of the BMD cutoff value.

Another consideration is that diagnostic criteria are based on BMD measures and cutoff values calculated for white women, so they may underestimate osteoporosis in minorities (CDC 2007d). Of note, a greater proportion of white women had hip-BMD–defined osteoporosis (88%), compared with the proportion of white women with the reported osteoporosis outcome measure (75%). It is also interesting that white race was significantly associated with decreased BMD using multiple linear regression but not with BMD-cutoff–defined osteoporosis using multiple logistic regression. Notwithstanding the limitations of linear regression for the nonparametric continuous primary predictor Cd, the discrepancy in significance of the dichotomous race covariate may suggest limited application of cutoff values to nonwhites. This possibility is strengthened by the additional discrepancy that white race was significantly associated with increased odds for self-reported osteoporosis, but not osteoporosis defined by BMD cutoff, and merits further research.

In this study we did not address fracture outcomes; however, it is noteworthy that associations between environmental Cd exposure and fracture risk have been previously reported in the literature. Staessen et al. (1999) found a statistically significant association between a 2-fold increase in U-Cd and 73% increased risk of fractures in women. The OSCAR study reported evidence of an association between increasing Cd exposure and excess risk of forearm fractures in people ≥50 years of age (Jarup and Alfven 2004). Alfven et al. (2004) found that the fracture hazard ratio increased by 18% per 1-nmol Cd/mmol creatinine for this same age group. Zhu et al. (2004) reported increased fracture prevalence among people ≥50 years of age living in a Cd-polluted area in China compared with residents of a control area.

A biological mechanism for Cd-induced osteoporosis is uncertain. It has been hypothesized that Cd-induced renal tubular damage leads to hypercalciuria, which secondarily causes bone mineral loss (Kjellstrom 1992; Satarug and Moore 2004). Other possible mechanisms underlying the effects of Cd on bone include interference with calcium absorption from the intestines, disruption of parathyroid hormone’s stimulation of vitamin D production in renal cells, and diminished kidney enzymatic action to activate vitamin D (Kjellstrom 1992). Yet, recent research has provided evidence of primary effects on bone (Akesson et al. 2006; Satarug and Moore 2004; Schutte et al. 2008). Akesson et al. (2006) interpreted findings of a positive association between U-Cd and deoxypyridinoline, a biomarker of bone resorption, to suggest a direct effect of Cd on bone in a Swedish population-based study of women. A Belgian population-based analysis of women without renal tubular dysfunction, as measured by the biomarker urinary retinol-binding protein, showed a positive association between U-Cd and two biomarkers of bone resorption, urinary hydroxylysylpyridinoline and lysylpyridinoline (Schutte et al. 2008).

A limitation of this study was our inability to adjust for biomarkers of renal tubular impairment in the primary analysis and to precisely adjust for Cd-induced tubular kidney damage in the secondary analysis, in which participant-reported physician diagnosis of kidney disease provided a weak surrogate relative to tubular dysfunction biomarkers such as retinol-binding protein (Schutte et al. 2008) and β_2_-microglobulin (Chen et al. 2006). Consequently, our analysis did not provide evidence to support conclusions regarding the effects of Cd on osteoporosis independent of renal tubular dysfunction. However, our findings of increased odds for osteoporosis at Cd levels below those previously associated with renal tubular dysfunction indicate the possibility of a direct effect. Moreover, Schutte et al. (2008) reported findings of direct effects of Cd on bone, independent of renal tubular dysfunction biomarkers. Further work is needed to understand the independent effects of Cd on osteoporosis and renal tubular dysfunction.

An additional limitation of our study was the potential for selection bias. NHANES conducts household surveys and does not include the institutionalized U.S. population. Colon-Emeric et al. (2007) estimated the prevalence of osteoporosis among nursing home residents at 50% of males and 64–90% of females. Because of missing data for this institutionalized elderly population, our findings omit measures of association between Cd and osteoporosis for this high-risk subpopulation. Moreover, NHANES III–examined sample response rates were lowest for women ≥ 80 years of age, so again, the frail elderly population may be underrepresented. Another limitation of this study is that Cd measurements were based on a single urine sample that does not capture possible significant temporal variations in U-Cd levels among NHANES participants. We adjusted U-Cd for urinary creatinine in an attempt to correct for varying concentrations among samples collected from different individuals at different times. Specific gravity has also been recommended for such an adjustment (Suwazono et al. 2005), but this variable was not available in NHANES. Although U-Cd adjusted for creatinine may be affected by age, gender, and body size (Suwazono et al. 2005), we controlled for these influences, and adjustment for creatinine is commonly used, allowing comparisons among a number of studies. Other limitations are lack of inclusion of other factors that may be associated with the risk of osteoporosis, such as menopause, hormone replacement therapy, adult calcium or vitamin D intake, excessive alcohol intake, and personal or family history of fracture.

## Conclusion

The present study suggests that U.S. women are at risk for osteoporosis at U-Cd levels below the U.S. Occupational Safety and Health Administration’s (OSHA) minimum safety standard of 3 μg/g (OSHA 2005) and even at levels < 1 μg/g, a concentration not associated with renal tubular damage. The cross-sectional design of NHANES limits determinations of causal associations, and we did not address the onset of osteoporosis in this study. U-Cd is a biomarker of long-term Cd exposure, however, and a temporal sequence between Cd exposure and osteoporosis is plausible. This national sample allows calculation of an attributable fraction for the entire population. Given that 73% of U.S. women ≥ 50 years of age are estimated to have Cd body burdens that are associated with excess risk (> 0.50 μg/g creatinine), as estimated from the NHANES III data set, these results suggest that 21% of osteoporosis prevalence among women ≥ 50 years of age may be attributable to Cd. Because these effects have been observed at current U.S. dietary exposures that constitute only 21% of PTWI for Cd (Egan et al. 2007), there is a need to investigate the relationship between Cd and osteoporosis and to reexamine the safe levels of Cd in food, the most common source of Cd exposure for the general population.

## Figures and Tables

**Table 1:** Descriptive statistics and unadjusted ORs for osteoporosis,^a^ women 50–90 years of age, NHANES III (1988–1994).

	All women (*n* = 3,207)		Osteoporosis (*n* = 664)		No osteoporosis (*n* = 2,543)		
Variable	No.	Percent	No.	Percent	No.	Percent	Unadjusted OR (*p*-value)
U-Cd levels (μg/g)							
≤0.50	870	27	131	20	739	29	
0.50–1.00	1,201	37	260	39	941	37	1.55 (< 0.01)
> 1.00	1,136	35	273	41	863	34	1.77 (< 0.01)
U-Cd arithmetic mean (μg/g)	0.96 (range, 0.007–16.17)		1.12 μg/g		0.92 μg/g		1.38^b^ (< 0.01)
Age, arithmetic mean (years)	67 (range, 50–90)		75 years		65 years		1.09 (< 0.01)
Race							
Nonwhite	764	24	82	12	682	27	
White	2,443	76	582	88	1,861	73	1.53 (0.01)
Income category, arithmetic mean^c^	16.87 (range, 1–27)		15.68		17.18		0.96 (< 0.01)
Smoking							
Never smoker	1,971	61	423	64	1,548	61	
Ever smoker	1,236	39	241	36	995	39	0.92 (0.41)
Underweight^d^ (< 127 lb or 57.61 kg)							
No	2,618	82	275	41	2,343	92	
Yes	579	18	388	59	191	8	8.03 (< 0.01)

a Osteoporosis defined per WHO hip BMD criteria (Looker et al. 1997).

b OR for overall U-Cd interpreted per 1-μg/g creatinine increment.

c Some respondents reported not knowing their income or did not provide an answer regarding their income category; we coded these data as missing. As a result, 84 subjects with osteoporosis and 292 subjects without osteoporosis had missing values.

d Weight was not recorded in the NHANES III data set for all respondents. We coded nonrecorded data as missing; as a result, one subject with osteoporosis and nine subjects without osteoporosis had missing values.

**Table 2:** Descriptive statistics and unadjusted ORs for osteoporosis,^a^ women 50–85 years of age, NHANES 1999–2004.

	All women (*n* = 1,051)		Osteoporosis (*n* = 200)		No osteoporosis (*n* = 851)		
Variable	No.	Percent	No.	Percent	No.	Percent	Unadjusted OR (*p*-value)
U-Cd levels (μg/g)							
≤0.50	527	50	87	44	440	52	
0.50–1.00	368	35	80	40	288	34	1.60 (0.04)
> 1.00	156	15	33	17	123	14	1.68 (0.05)
U-Cd arithmetic mean (μg/g)	0.63 (range, <0.01^b^–4.199)		0.69 μg/g		0.62 μg/g		1.59^c^ (0.02)
Age, arithmetic mean (years)	67 (range, 50–85)		71 years		66 years		1.05 (< 0.01)
Race							
Nonwhite	459	44	50	25	409	48	
White	592	56	150	75	442	52	2.47 (< 0.01)
Income category, arithmetic mean^d^	5.759 (range, 1–11)		5.527		5.816		0.92 (0.02)
Smoking							
Never smoker	620	59	110	55	510	60	
Ever smoker	431	41	90	45	341	40	1.39 (0.08)
Underweight (< 127 lb or 57.61 kg)							
No	868	83	149	75	719	84	
Yes	183	17	51	25	132	16	2.02 (< 0.01)
Renal impairment^e^							
No	1,004	96	186	93	818	97	
Yes	43	4	14	7	29	3	2.92 (0.02)

a Osteoporosis defined per self-report of physician diagnosis.

b NHANES assigned U-Cd fill values of 0.03 and 0.04 ng/mL for first and second fill value of limits of detection, respectively; dividing by mg/dL creatinine yielded values < 0.01.

c OR for overall U-Cd interpreted per 1-μg/g creatinine increment.

d Some respondents reported not knowing their income or did not provide an answer regarding their income category; we coded these data as missing. As a result, 16 subjects with osteoporosis and 106 subjects without osteoporosis were missing values for income.

e Some respondents reported not knowing whether a doctor told them they have renal impairment or did not provide an answer. We coded these data as missing. As a result, four observations subjects with renal impairment without osteoporosis had missing values.

**Table 3:** U-Cd (μg/g creatinine)-adjusted ORs for osteoporosis by outcome measure (data source), women ≥ 50 years of age.

	Osteoporosis				Satterwaithe *p*-value
	Yes	No	OR	95% CI	
Hip BMD (NHANES III, 1988–1994)^a^					
Overall U-Cd	579	2,247	1.15^b^	1.00–1.33	0.05
≤0.50 μg/g	110	643	1.00		
0.50–1.0 μg/g	228	839	1.43	1.02–2.00	0.04
>1.0 μg/g	241	765	1.40	0.97–2.03	0.07
Respondent-reported physician-diagnosed osteoporosis (NHANES 1999–2004)^c^					
Overall U-Cd	184	741	1.65^b^	1.07–2.54	0.02
≤0.50 μg/g	82	386	1.00		
0.50–1.0 μg/g	69	250	1.46	0.84–2.55	0.18
>1.0 μg/g	33	105	1.47	0.81–2.66	0.20

a Adjusted for age, race, income, ever-smoker, and underweight.

b OR for overall U-Cd interpreted per 1-μg/g creatinine increment.

c Adjusted for age, race, income, ever-smoker, underweight, and survey-respondent–reported physician diagnosis of renal impairment.

**Table 4:** U-Cd (μg/g creatinine)-adjusted ORs^a^ for osteoporosis by outcome measure (data source), women ≥50 years of age, by smoking status.

	Osteoporosis				Satterwaithe *p*-value
	Yes	No	OR	95% CI	
Hip BMD (NHANES III, 1988–1994)^b^					
Never-smokers	360	1,361	1.20	0.98–1.48	0.08
Ever-smokers	219	886	1.12	0.90–1.39	0.29
Respondent-reported physician-diagnosed osteoporosis (NHANES 1999–2004)^c^					
Never-smokers	100	441	2.05	0.99–4.23	0.05
Ever-smokers	84	300	1.55	0.91–2.65	0.10

a ORs interpreted per 1-μg/g creatinine increment.

b Adjusted for age, race, income, and underweight.

c Adjusted for age, race, income, underweight, and survey-respondent–reported physician diagnosis of renal impairment.

**Table 5:** Multiple linear regression results for dependent variables femur BMD^a^ (multiple *R*^2^ = 0.31) and total hip BMD^b^ (multiple *R*^2^ = 0.32), women 50–90 years of age, NHANES III (1988–1994).

	β-Coefficient		95% CI		*p*-Value (Satterwaithe-adjusted *F*)	
Independent variables	Femur	Total hip	Femur	Total hip	Femur	Total hip
Log (Cd μg/g creatinine + 1)	−0.04	−0.05	−0.06 to −0.02	−0.07 to −0.03	< 0.01	< 0.01
Age	−0.01	−0.01	−0.01 to −0.00	−0.01 to −0.01	< 0.01	< 0.01
White race	−0.07	−0.05	−0.09 to −0.05	−0.07 to −0.03	< 0.01	< 0.01
Income	0.00	0.00	0.00 to 0.00	0.00 to 0.00	0.96	0.73
Smoking	0.00	−0.01	−0.02 to 0.01	−0.03 to 0.01	0.59	0.28
Underweight^c^	−0.11	−0.13	−0.12 to −0.09	−0.15 to −0.11	< 0.01	< 0.01

a BMD (g/cm^2^) of femoral neck.

b BMD (g/cm^2^) of total hip.

c < 127 lb (57.61 kg).
